# Exploring the In Vivo Anti-Inflammatory Actions of Simvastatin-Loaded Porous Microspheres on Inflamed Tenocytes in a Collagenase-Induced Animal Model of Achilles Tendinitis

**DOI:** 10.3390/ijms19030820

**Published:** 2018-03-12

**Authors:** Chandong Jeong, Sung Eun Kim, Kyu-Sik Shim, Hak-Jun Kim, Mi Hyun Song, Kyeongsoon Park, Hae-Ryong Song

**Affiliations:** 1 Department of Orthopedic Surgery and Rare Diseases Institute, Korea University College of Medicine, Guro Hospital, Guro-dong, Guro-gu, Seoul 08308, Korea; cdjeong85@gmail.com (C.J.); sekim10@korea.ac.kr (S.E.K.); dakjul@korea.ac.kr (H.-J.K.); wwiiw@naver.com (M.H.S.); 2 Department of Biomedical Science, Korea University College of Medicine, Anam-dong, Seongbuk-gu, Seoul 02841, Korea; breakdown88@nate.com; 3 Department of Systems Biotechnology, Chung-Ang University, Anseong, Gyeonggi-do 17546, Korea

**Keywords:** Achilles tendinitis, simvastatin, porous microspheres, anti-inflammation, tendon healing

## Abstract

Tendon rupture induces an inflammatory response characterized by release of pro-inflammatory cytokines and impaired tendon performance. This study sought to investigate the therapeutic effects of simvastatin-loaded porous microspheres (SIM/PMSs) on inflamed tenocytes in vitro and collagenase-induced Achilles tendinitis in vivo. The treatment of SIM/PMSs in lipopolysaccharide (LPS)-treated tenocytes reduced the *mRNA* expressions of pro-inflammatory cytokines (Matrix metalloproteinase-3 (*MMP-3*), cyclooxygenase-2 (*COX-2*), interleukin-6 (*IL-6*), and tumor necrosis factor-α (*TNF-α*)). In addition, the local injection of SIM/PMSs into the tendons of collagenase-induced Achilles tendinitis rat models suppressed pro-inflammatory cytokines (*MMP-3*, *COX-2*, *IL-6*, *TNF-α*, and *MMP-13*). This local treatment also upregulated anti-inflammatory cytokines (*IL-4*, *IL-10*, and *IL-13*). Furthermore, treatment with SIM/PMSs also improved the alignment of collagen fibrils and effectively prevented collagen disruption in a dose-dependent manner. Therefore, SIM/PMSs treatment resulted in an incremental increase in the collagen content, stiffness, and tensile strength in tendons. This study suggests that SIM/PMSs have great potential for tendon healing and restoration in Achilles tendinitis.

## 1. Introduction

Most acute and chronic tendon injuries are the result of gradual wear and tear to the tendon from either overuse or aging. Tendon problems develop both in athletes and in the general population [1]. Achilles tendinitis is an inflammatory injury characterized by chronic pain and swelling [2]. Injured tendons induce a local inflammation response, which is mediated by the release of inflammation markers including pro-inflammatory cytokines (e.g., interleukin-1β (IL-1β), tumor necrosis factor-α (TNF-α)), matrix metalloproteinases (MMPs; MMP-2, -3, -9, and -13, etc.) and cyclooxygenase-2 (COX-2). Eventually, there is cell damage and/or death as well as disintegration of the extracellular matrix (ECM) at the injured sites. Ultimately, these changes decrease the biomechanical properties (i.e., tensile strength) of the tendon [3,4,5,6]. Given its inflammatory nature, non-steroidal anti-inflammatory drugs (NSAIDs) are one of the treatment approaches for Achilles tendinitis. However, previous studies have found that the long-term use of oral NSAIDs can have adverse side effects, such as gastrointestinal bleeding, ulceration, and perforation [7]. In contrast, the local application of NSAIDs effectively reduced pain and swelling without many of these adverse events; however, their long-term effects are minimal [8,9].

Statins as 3-hyroxy-3-methylglutaryl coenzyme A (HMG-CoA) reductase inhibitors have pleiotropic effects, including anti-inflammatory, antioxidant, immune-modulatory, and angiogenesis stimulation effects [10,11]. Previous studies have found that statins inhibit the secretion of IL-6 and TNF-α in co-cultured human vascular smooth muscle cells (SMCs) and human mononuclear cells (MNCs) or macrophages [12,13]. Despite their positive effects, statins also have adverse effects on muscle, including myalgias, myositis, rhabdomyolysis, and myopathies [14,15,16]. Eliasson et al. recently reported that simvastatin and atorvastatin have negative effects on tendons due to the decreased mechanical properties of tendon constructs and catabolic changes in the *gene* expression pattern [17]. These adverse effects mainly occur in the setting of high statin doses.

In order to overcome these adverse effects and to investigate the enhanced therapeutic effects of Achilles tendinitis, injectable drug delivery systems have been developed for local application. For instance, porous microspheres are suitable for the controlled and long-term delivery of drugs and proteins [18,19,20]. We recently developed a porous microsphere using a simple fluidic device and a poly(lactic-co-glycolic acid) (PLGA) polymer as the drug delivery carrier [18,19,20,21]. This PLGA polymer is non-toxic, biocompatible, and biodegradable. It is also the Food and Drug Administration (FDA)-approved polymer for clinical applications [22]. In this study, we prepared simvastatin-loaded porous PLGA microspheres (SIM/PMSs). We investigated whether SIM/PMS systems showed anti-inflammatory effects in lipopolysaccharide (LPS)-treated tenocytes in vitro as well as in vivo anti-inflammation, tendon healing, and tissue restoration in a collagenase-induced Achilles tendinitis rat model.

## 2. Results

### 2.1. Characterizing PMSs and SIM/PMSs in an In Vitro Drug Release Study

**Figure 1:**
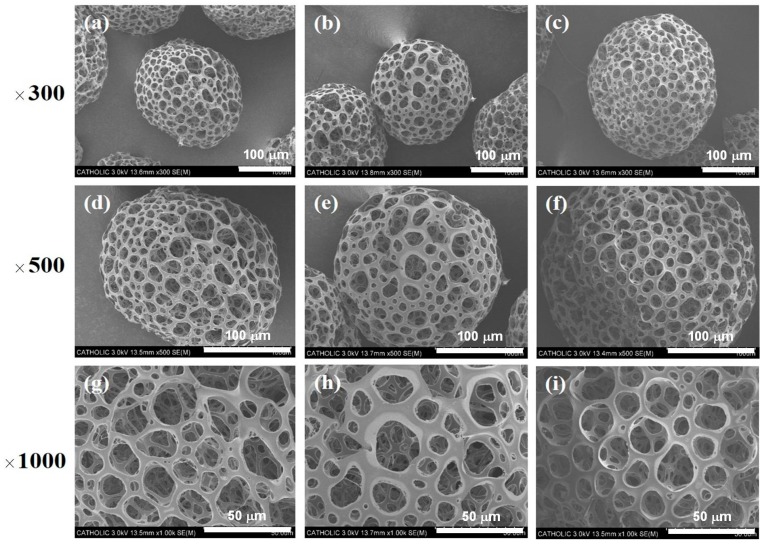
SEM images of porous microspheres (PMSs) (**a**,**d**,**g**), simvastatin (SIM) (1 mM)/PMSs (**b**,**e**,**h**), and SIM (5 mM)/PMSs (**c**,**f**,**i**).

**Figure 2:**
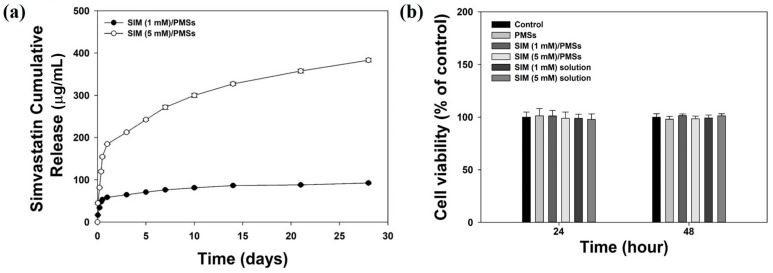
(**a**) In vitro release profiles of simvastatin from SIM (1 mM)/PMSs and SIM (5 mM)/PMSs. (**b**) Cytotoxicity of simvastatin (1 mM and 5 mM), PMSs, SIM (1 mM)/PMSs, and SIM (5 mM)/PMSs at 24 h and 48 h. Error bars represent the means ± SDs (*n* = 5).

The size, porosity, and shape of the prepared PMSs with or without simvastatin were analyzed using a scanning electron microscope (SEM). The prepared PMSs and two SIM/PMSs with different drug amounts had similar morphologies (Figure 1). The PMSs and SIM/PMSs were also similar in size, at approximately 250–300 μm in diameter. All of the PMSs and SIM/PMSs were highly porous structures. The pores were interconnected. The average pore sizes of the PMSs, SIM (1 mM)/PMSs, and SIM (5 mM)/PMSs were 25.37 ± 2.12 μm, 26.52 ± 3.49 μm, and 26.32 ± 2.08 μm, respectively. The loading amounts of simvastatin per 30 mg of PMSs were 143.51 ± 6.69 μg for SIM (1 mM)/PMSs and 730.88 ± 33.25 μg for SIM (5 mM)/PMSs. The in vitro release profiles of simvastatin from the SIM/PMSs are shown in Figure 2a. On the first day, there was 58.54 ± 0.98 μg of simvastatin released from SIM (1 mM)/PMSs and 184.31 ± 0.78 μg from SIM (5 mM)/PMSs. Over 28 days, the SIM (1 mM)/PMSs and SIM (5 mM)/PMSs released 92.39 ± 1.87 μg and 383.04 ± 4.13 μg of simvastatin, respectively.

### 2.2. In Vitro Cytotoxicity

Cytotoxicities for PMSs, simvastatin (1 mM or 5 mM), SIM (1 mM)/PMSs, and SIM (5 mM)/PMSs were measured using a CCK-8 assay kit (Dojindo Inc., Tokyo, Japan) at 24 h and 48 h. Cell viabilities in all groups were maintained at over 96%, indicating that PMSs, simvastatin, and SIM/PMSs had no toxic effects on tenocytes (Figure 2b).

### 2.3. Anti-Inflammatory Properties of SIM/PMSs in Inflamed Tenocytes

**Figure 3:**
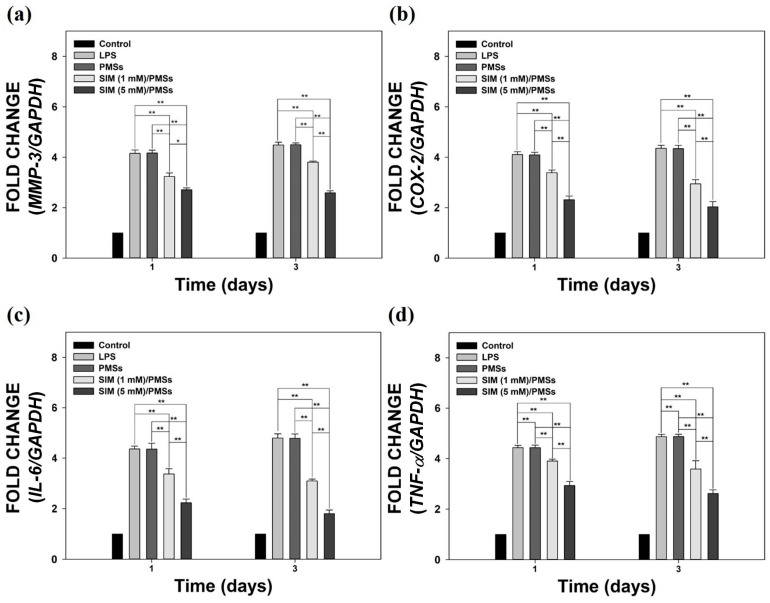
The relative *mRNA* expression levels of pro-inflammatory cytokines, including: (**a**) *MMP-3*, (**b**) *COX-2*, (**c**) *IL-6*, and (**d**) *TNF-α* in lipopolysaccharide (LPS)-stimulated tenocytes that were cultured with PMSs, SIM (1 mM)/PMSs, and SIM (5 mM)/PMSs on days 1 and 3. Error bars represent the means ± SDs (*n* = 5). (* *p* < 0.05 and ** *p* <0.01).

We sought to evaluate the in vitro anti-inflammatory effects of SIM/PMSs in LPS-stimulated tenocytes. The *mRNA* expression levels of pro-inflammatory cytokines, such as *MMP-3*, *COX-2*, *IL-6*, and *TNF-α*, were determined using real-time PCR on days 1 and 3 (Figure 3). The LPS-stimulated tenocytes had the highest *mRNA* levels of pro-inflammatory cytokines on days 1 and 3. The PMSs without simvastatin did not suppress the *mRNA* levels of pro-inflammatory cytokines as did those in the LPS-treated group. This finding suggests that the PMSs alone have no anti-inflammatory properties. However, the SIM/PMSs significantly and dose-dependently decreased the *mRNA* levels of *MMP-3*, *COX-2*, *IL-6*, and *TNF-α* in LPS-stimulated tenocytes compared to those in the other groups on days 1 and 3 (** *p* < 0.01). This decrease implies that the released simvastatin from the microspheres can suppress inflammatory responses in LPS-stimulated tenocytes.

### 2.4. Histopathological Evaluations

**Figure 4:**
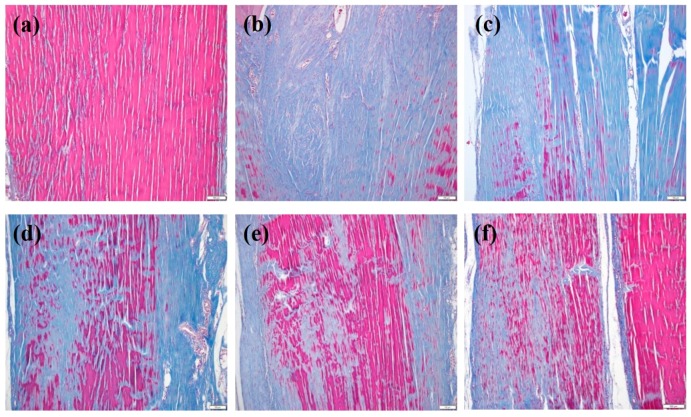
Masson’s trichrome staining at 7 weeks after collagenase (Col (I)) injection into tendon tissues, and at 6 weeks after treatment with PMSs, simvastatin, SIM (1 mM)/PMSs, and SIM (5 mM)/PMSs. Groups are categorized as follows: (**a**) control (no treatment); (**b**) Col (I); (**c**) Col (I) + PMSs; (**d**) Col (I) + simvastatin; (**e**) Col (I) + SIM (1 mM)/PMSs, and (**f**) Col (I) + SIM (5 mM)/PMSs. Scale bar: 100 μm. Red: collagen fibers; Blue: collagen matrix breakdown.

We developed collagenase-induced rat models of Achilles tendinitis. We used these animal models to confirm the in vivo suppression of tendon degeneration and anti-inflammatory responses of SIM/PMSs. Histopathological examination with Masson’s trichrome staining was performed to determine whether SIM/PMSs can prevent tendon disruption. As shown in Figure 4a, normal tendons had well-aligned collagen fiber organization and no tendon disruption. In contrast, collagenase injection led to severe collagen matrix breakdown with an absence of well-aligned collagen fibers (Figure 4b). In PMSs treatment alone, the severe collagen matrix breakdown was still shown, suggesting that PMSs have no preventative effects on collagen disruption (Figure 4c). However, treatment with simvastatin and SIM/PMSs was sufficient to suppress collagen matrix disruption (Figure 4d–f). The SIM/PMSs treated groups exhibited much more aligned collagen fiber organization and effectively prevented collagen disruption in a dose-dependent manner compared to that of simvastatin. This result suggests that SIM/PMSs have better tendon restoration effects than does simvastatin (Figure 4e,f).

### 2.5. In Vivo Tendon Restorative Effects of SIM/PMSs and Biomechanical Study

**Figure 5:**
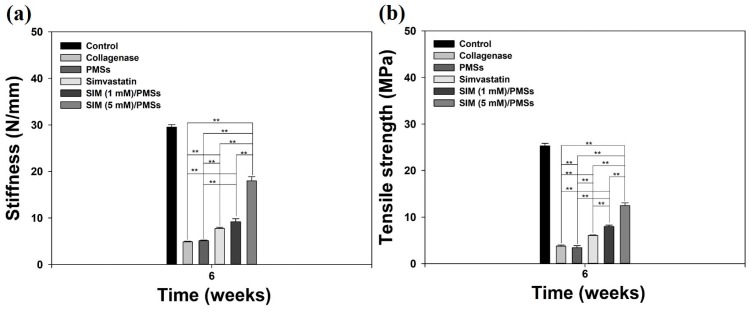
(**a**) Stiffness and (**b**) tensile strengths of Achilles tendon tissues isolated from collagenase-induced Achilles tendinitis rat models in each group at 6 weeks after treatments with PMSs, simvastatin, SIM (1 mM)/PMSs, and SIM (5 mM)/PMSs. Data represent means ± SDs (*n* = 4). (** *p* < 0.01).

**Figure 6:**
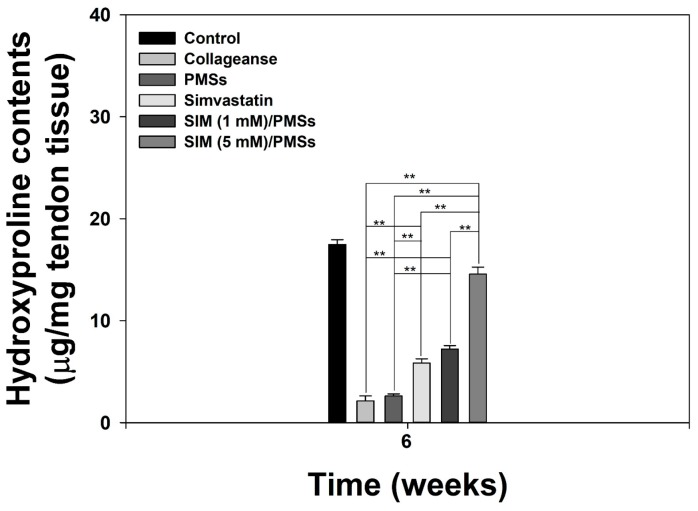
Hydroxyproline contents in Achilles tendon tissues from collagenase-induced Achilles tendinitis rat models 6 weeks after treatment with PMSs, simvastatin, SIM (1 mM)/PMSs, and SIM (5 mM)/PMSs. Data represent means ± SDs (*n* = 4), (** *p* < 0.01).

In order to further demonstrate the tendon restorative effects of SIM/PMSs, we performed biomechanical studies, including stiffness and tensile strengths, of tendon tissues. The stiffness and tensile strengths of the tendon tissues in the collagenase-treated group and PMSs-treated group were much lower than were those in the control (normal) group at 6 weeks (Figure 5). Simvastatin increased the stiffness and tensile strengths of the tendons compared to that of the collagenase-treated group and PMSs-treated group. However, the tendon healing effects of both SIM/PMSs groups were much more effective in a dose-dependent manner than were those of simvastatin (** *p* < 0.01). A hydroxyproline assay was performed at six weeks after the drug treatments in order to determine the change in collagen content in the Achilles tendinitis animal model. The hydroxyproline contents of the Achilles tendon in the collagenase-treated group and PMSs-treated group were significantly lowered than were those in the control (normal) group (Figure 6). Treatments with simvastatin and two SIM/PMSs increased the hydroxyproline content significantly more than did treatment in the collagenase-treated group and PMSs-treated group (** *p* < 0.01). The SIM/PMSs-treated groups also had much higher hydroxyproline content in a dose-dependent manner than did the simvastatin-treated group (** *p* < 0.01).

### 2.6. In Vivo Anti-Inflammatory Effects of SIM/PMSs

**Figure 7:**
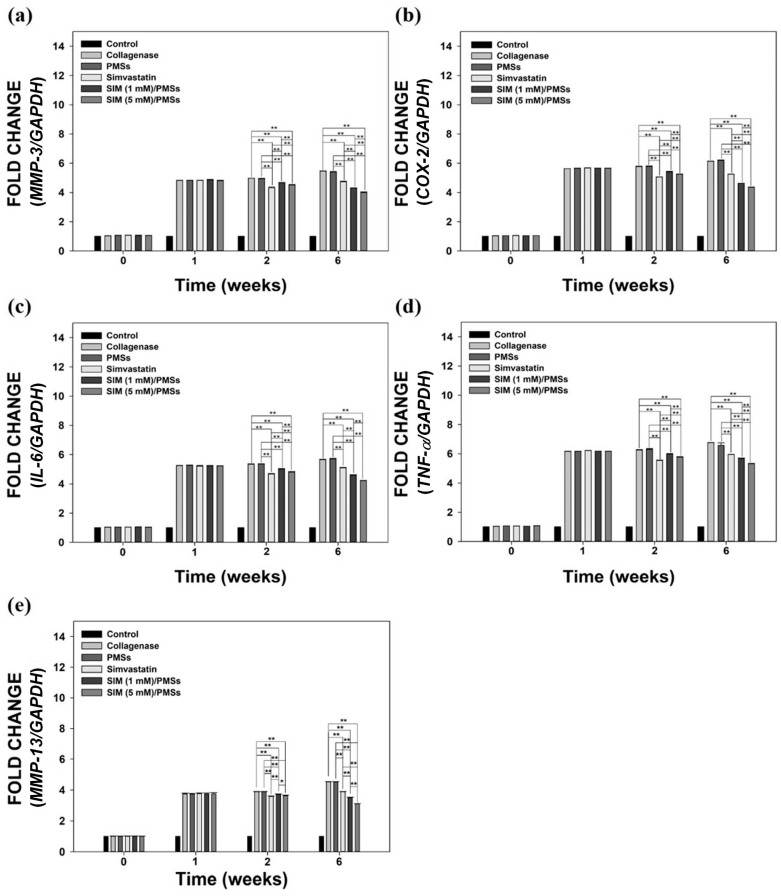
The relative *mRNA* expression levels of pro-inflammatory cytokines, including: (**a**) *MMP-3*; (**b**) *COX-2*, (**c**) *IL-6*, (**d**) *TNF-α*, and (**e**) *MMP-13* in whole blood samples from a collagenase-induced Achilles tendinitis rat model in each group. Whole blood samples were collected from collagenase-induced Achilles tendinitis rat models in each group at 0, 1, 2, and 6 weeks after treatments with PMSs, simvastatin, SIM (1 mM)/PMSs, and SIM (5 mM)/PMSs. Each *mRNA* expression level was determined using real time-PCR analysis. Data represent means ± SDs (*n* = 4), (* *p* < 0.05 and ** *p* < 0.01).

**Figure 8:**
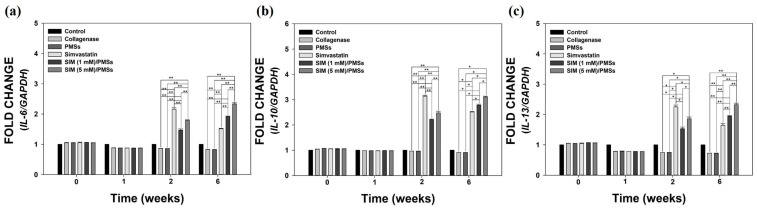
The relative *mRNA* expression levels of anti-inflammatory cytokines, including: (**a**) *IL-4*, (**b**) *IL-10*, and (**c**) *IL-13* in whole blood samples from the collagenase-induced Achilles tendinitis rat model in each group. Whole blood samples were collected from the rat models in each group at 0, 1, 2, and 6 weeks after treatment with PMSs, simvastatin, SIM (1 mM)/PMSs, and SIM (5 mM)/PMSs. Each *mRNA* expression level was determined using real time-PCR analysis. Data represent means ± SDs (*n* = 4), (* *p* < 0.05 and ** *p* < 0.01).

We next sought to further investigate the in vivo anti-inflammatory effects of SIM/PMSs in collagenase-induced Achilles tendinitis rat models. The *mRNA* levels of pro-inflammatory cytokines (*MMP-3*, *COX-2*, *IL-6*, *TNF-α*, and *MMP-13*), as well as anti-inflammatory cytokines (*IL-4*, *IL-10*, and *IL-13*), were measured in whole blood samples collected from the rats in each group. Figure 7 shows that the *mRNA* levels of all of pro-inflammatory cytokines in the collagenase-treated group were significantly upregulated (up to over 5-fold) compared to those in the control (normal) group. There were no significant differences in the *mRNA* levels between the collagenase-treated group and PMSs-treated group. At two weeks, the *mRNA* levels of pro-inflammatory cytokines in the two SIM/PMSs-treated groups were similar or slightly higher than those in the simvastatin-treated groups. However, at six weeks, the *mRNA* levels in both SIM/PMSs-treated groups were much lower than were those in the simvastatin-treated group, in a dose-dependent manner (** *p* < 0.01). The *mRNA* levels of anti-inflammatory cytokines (*IL-4*, *IL-10*, and *IL-13*) in the collagenase-treated group (or PMSs-treated groups) were similarly maintained or slightly lower than were those in the control (normal) group during the 6 weeks (Figure 8). In contrast, groups treated with simvastatin (the real treated dose; 105 μg/rat), SIM (1 mM)/PMSs (the real treated dose; 2.39 μg/rat), and SIM (5 mM)/PMSs (the real treated dose; 12.18 μg/rat) had increased *mRNA* levels of anti-inflammatory cytokines. Simvastatin had much higher levels than did the two SIM/PMSs-treated groups at two weeks. This result can be explained by the fact that the treated dose of simvastatin was much higher than were those of the two SIM/PMSs groups. However, the two SIM/PMSs-treated groups displayed much higher *mRNA* levels of anti-inflammatory cytokines in a dose-dependent manner than did the simvastatin-treated group at 6 weeks (** *p* < 0.01). These data suggest that SIM/PMSs were more effective than was simvastatin at suppressing the inflammatory responses induced by collagenase injection into tendon tissues. This result is mostly likely because SIM/PMSs gradually released simvastatin molecules from the microspheres over a long period of time. Simvastatin is a small molecular weight molecule that may passively cross into the blood stream and be easily excreted from local treatment sites. Therefore, the simvastatin group may have experienced a shorter exposure period to the simvastatin than did the SIM/PMSs groups.

## 3. Discussion

Achilles tendinitis occurs in both active and inactive individuals due to overuse or aging. Achilles tendinitis is accompanied by pain, swelling, and impaired tendon function in its early stage [23]. It eventually can lead to partial or total tendon rupture. Tendon disruption and injury provokes local inflammation responses by inducing pro-inflammatory cytokines, MMPs, and ECM disintegration [6]. Ultimately, this inflammation impairs the biomechanical properties of the tendon tissues. Prior reports have suggested that statins, 3-hydroxy-3-methylglutaryl coenzyme A (HMG-CoA) reductase inhibitors, have pleiotropic effects including anti-inflammation, anti-oxidation, and immune-modulation effects [24,25]. Retrospective studies have reported that there are double-sided characteristics on statin-associated tendon disorders. Simvastatin may have a protective role in tendinopathies in patients with severe hyperlipidemia [26,27,28]. Treatment with statins may also enhance tendon healing through stimulation of the COX-2/prostaglandin E2 receptor 4 (PGE2 EP4) pathway. Statins may also enhance anterior cruciate ligament (ACL) healing through their effects on angiogenesis and osteogenesis [29]. In contrast, other studies have suggested that several common side effects of statins (i.e., muscle weakness and pain) actually increase the risk of tendinopathy and rupture [30,31] and tendon complications [26,30,32]. However, previous in vivo studies showed that statin treatment reduced the mechanical properties of tendon constructs and increased the *gene* expressions of *MMPs* (such as *MMP-1*, *MMP-3*, and *MMP-13*) in the ECM of tendons [31,32]. Ultimately, there remains active controversy regarding statins’ role as either detrimental or beneficial to tendon healing.

In this study, we developed simvastatin-loaded porous microspheres (SIM/PMSs). These microspheres were built using a simple fluidic device consisting of a discontinuous phase channel (homogenized gelatin and PLGA solution with or without simvastatin) and a continuous phase channel (as previously described by our group) [18,19,20,21]. Simple fluidic devices are very useful to control the size and porosity of the microspheres. The prepared PMSs, with or without simvastatin, were spherical shapes approximately 250–300 μm in diameter. Their pores were interconnected and 25–30 μm in size. Hydrophobic simvastatin could be readily encapsulated within hydrophobic PLGA porous microspheres. Initially, the SIM/PMSs allowed fast drug release given the diffusion from the surface of highly porous microspheres. However, the release of simvastatin from the microspheres was incomplete over the four weeks given polymer degradation. This result suggests that SIM/PMSs are suitable for long-term drug delivery.

The in vitro anti-inflammatory effects of PMSs were investigated using tenocytes, because they are fibroblast-like differentiated cells found throughout the tendon structure. Tenocytes synthesize ECM and induce the assembly of early collagen fibers (as the basic units of the tendon). The in vitro inflammatory environment was mimicked by treating LPS to the tenocytes. As previously reported, the LPS-treated cells increased their expression of certain cytokines, including TNF-α, IL-6, and IL-1β. These cytokines further stimulate tenocytes to induce pro- and anti-inflammatory cytokines, including TNF-α, IL-6, IL-10, IL-1β, COX-2, and ECM-degrading enzymes, such as MMP-1, -3, and -13 [33,34]. Previous studies have reported that statins have anti-inflammatory activities on several cells. For example, LPS-stimulated macrophages that were exposed to statins decreased their expression of IL-6 and TNF-α [12,35]. Statins also inhibited macrophage production of IL-6, IL-8, MMP-1, -3, and -9 [36,37]. Consistent with the findings of previous studies, we also demonstrated that treatment of LPS-treated tenocytes with PMSs alone does not reduce the *mRNA* levels of pro-inflammatory cytokines (i.e., *MMP-3*, *COX-2*, *IL-6*, and *TNF-α*). In contrast, SIM/PMSs could significantly decrease their *mRNA* levels in a dose-dependent manner. These data suggest that SIM/PMSs suppress inflammation responses by downregulating the *mRNA* expression levels of multiple pro-inflammatory cytokines in inflamed tenocytes.

Injection of collagenase type I consistently leads to tendon disruption accompanied by an inflammatory response [38]. Therefore, collagenase injection serves as an adequate method of developing an Achilles tendinitis model. In particular, injection of collagenase into a tendon disrupts collagen fibers, changes the biochemical and biomechanical properties of the tendon, and more closely resembles the main histopathologic features and dysfunctions of human tendinopathy [38]. Therefore, we used a collagenase-induced Achilles tendinitis rat model to study the in vivo anti-inflammatory and tendon-healing effects of SIM/PMSs. Under histological examination with Masson’s trichrome staining, we found that tendon treatment with collagenase led to destruction of the well-aligned collagen fiber organization and severe collagen matrix breakdown. The PMSs did not prevent collagen matrix disruption. In contrast, SIM/PMSs effectively decreased the collagen disruption and repaired collagen organization in a dose-dependent manner. Although simvastatin also prevented collagen matrix disruption, its therapeutic effect is lower than those of the two SIM/PMSs groups. Previous study reported that an increase in the hydroxyproline content is directly correlated to early maturation of fibroblasts, early parallel arrangement of collagen fibers, and bundle formation [39]. This group also found that there is a close relationship between the absolute amount of collagen and mechanical strength. In this study, the local treatment of SIM/PMSs on collagenase-treated tendon tissues had beneficial effects on the healing tendon. SIM/PMSs enhanced the hydroxyproline content of the treated tendon tissues more so than did collagenase. This result correlated with increased collagen fibrin organization using Masson’s trichrome stain. The collagen content increased after SIM/PMS treatment. Therefore, the stiffness and tensile strength of tendon tissues in the SIM/PMS-treated groups were markedly enhanced compared to those of the other groups (including collagenase, PMSs, and simvastatin-treated groups). These data suggest that using SIM/PMSs as a long-term simvastatin delivery system is useful for restoration of the tendon tissues in collagenase-induced Achilles tendinitis.

Tendon injuries (i.e., ruptures or tendinopathy) typically heal in three different phases. These are the inflammatory phase, proliferative phase or collagen-producing phase, and finally the remodeling phase [40,41]. During this healing process, the modulation of multiple pro- and anti-inflammatory cytokines plays an important role in improving tendon healing [42]. Excessive pro-inflammatory cytokines provoke inflammatory reactions, thereby leading to cell damage and ECM disintegration at injured sites [6]. In contrast, anti-inflammatory cytokines attract fibroblasts to restore the injured tissues [43]. In order to investigate the in vivo anti-inflammatory effects of SIM/PMSs on collagenase-induced Achilles tendinitis models, we monitored the *mRNA* levels of both pro-inflammatory cytokines (*MMP-3*, *COX-2*, *IL-6*, *TNF-α*, and *MMP-13*) and anti-inflammatory cytokines (*IL-4*, *IL-10*, and *IL-13*). We made these measurements from the blood at scheduled time points, because an indirect assessment of inflammatory markers in the blood can support the hypothesis of local inflammation at injured sites [44,45]. At 1, 2, and 6 weeks after collagenase injection, the *mRNA* levels of the pro-inflammatory cytokines were significantly increased. In contrast, the *mRNA* levels of anti-inflammatory cytokines did not increase. Compared to the collagenase-treated group, PMSs alone did not affect the *mRNA* expression levels of pro-inflammatory cytokines or anti-inflammatory cytokines, indicating that PMSs have no anti-inflammatory activity. In contrast, treatment with simvastatin and SIM/PMSs can downregulate pro-inflammatory cytokines and upregulate anti-inflammatory cytokines. However, the therapeutic effects of SIM/PMSs were superior to those of simvastatin.

This study demonstrates that SIM/PMSs have many beneficial therapeutic effects, including anti-inflammation and tendon healing effects, on a collagenase-induced Achilles tendinitis rat model. Interestingly, treatment with SIM/PMSs as a long-term simvastatin delivery system had much better therapeutic efficacy than did simvastatin alone. As our group has previously described, the biodegradation of microspheres (as a long-term drug delivery system) can be tailored from a few weeks to several months depending on the polymer compositions (such as the ratio of poly(lactic acid) to poly(glycolic acid)). These compositions ultimately influence the drug release rates from the drug delivery system [21]. Treatment with high doses of simvastatin alone also had positive effects on Achilles tendinitis at first. However, its therapeutic effects are short-lived, because this small molecular drug eventually passively diffuses into the blood stream and away from the injured sites. In order to achieve good therapeutic effects with a small molecular drug, therefore, repeated injections would be needed. Regardless, side effects and toxicities must be considered. Moreover, our study has some limitations, including the lack of *mRNA* expression levels for pro- and anti-inflammatory cytokines in tissues as well as no pharmacokinetic data of simvastatin and SIM/PMSs after local treatments. Despite these limitations, our study showed that using SIM/PMSs as a long-term delivery system will have a great potential to suppress inflammatory responses and enhance healing and restoration of tendon tissues.

## 4. Materials and Methods

### 4.1. Materials

PLGA (50:50; Resomer^®^ RG505) was supplied by Boehringer Ingelheim (Ingelheim, Germany). Poly vinyl alcohol (PVA, molecular weight: 13,000–23,000, 98% hydrolyzed), dichloromethane (DCM), gelatin from porcine skin, simvastatin (SIM), and LPS were obtained from Sigma-Aldrich (St. Louis, MO, USA). Cellulose-ester dialysis membrane (MWCO; 6–8 kDa) was obtained from Spectrum Laboratories (Milpitas, CA, USA). Dulbecco’s modified Eagle’s medium (DMEM), fetal bovine serum (FBS), phosphate-buffered saline (PBS), and penicillin-streptomycin were obtained from Gibco (Rockville, MD, USA).

### 4.2. Fabrication of SIM-Loaded Porous Microspheres (SIM/PMSs)

In order to fabricate SIM/PMSs, a fluidic device method was used, as previously described [18,19,20,21]. Briefly, PLGA (2 weight % (wt %)), with or without simvastatin (1 or 5 mM), was dissolved in DCM (7 mL). Gelatin (7.5 wt %) and PVA (2 wt %) were dissolved in deionized water (DW, 10 mL). Next, the gelatin (3 mL) and PVA (0.5 mL) solutions were added to the PLGA solution with or without SIM. These mixtures were emulsified with a homogenizer (Ultra-Turrax T-25 Basic, IKA, Woonsocket, RI, USA) at 13,500 rpm for 1 min. The emulsified solution was introduced into the discontinuous phase. Another PVA (2 wt %) solution was prepared as a continuous phase. In order to make PMSs, the continuous phase and discontinuous phase flow rates were 0.5 mL/min and 0.05 mL/min, respectively. The emulsion droplets were placed in warm water (45 °C) and gently stirred for 24 h to remove any residual gelatin within the microspheres and DCM. The fabricated microspheres were washed three times with warm DW, collected, and lyophilized for three days. The PMSs are designated according to the amount of simvastatin as PMSs (no simvastatin), SIM (1 mM)/PMSs, and SIM (5 mM)/PMSs, respectively.

### 4.3. Characterization of PMSs and SIM/PMSs

The morphologies of the PMSs, SIM (1 mM)/PMSs, and SIM (5 mM)/PMSs were observed using scanning electron microscopy (SEM, S-2300, Hitachi, Tokyo, Japan). Each sample was coated with platinum (Pt) using a sputter coater (Eiko IB, Tokyo, Japan), and examined at an accelerated voltage of 3 kV. The average pore sizes of randomly selected microspheres in each group (*n* = 50 pores/group) were determined using Image J (Ver. 1.2, Bethesda, MD, USA) based on the SEM images.

In order to determine the loading amount of simvastatin within SIM (1 mM)/PMSs and SIM (5 mM)/PMSs, 30 mg of each sample was dissolved in DCM. The loading amount of SIM was analyzed at 250 nm using the UV/Vis spectrophotometer (UV-1800, Shimadzu, Kyoto, Japan).

### 4.4. In Vitro Drug Release Study

In order to evaluate in vitro simvastatin release from the microspheres, the samples (10 mg) in each group were dispersed in a 50 mL conical tube including 1 mL PBS solution (pH 7.4) as the release medium. This mixture was gently shaken at a rate of 100 rpm in a shaking water bath at 37 °C. At the pre-determined time intervals, the PBS was replaced with fresh PBS medium. The amount of simvastatin that was released from the microspheres was determined at 250 nm using the UV/Vis spectrophotometer.

### 4.5. In Vitro Cytotoxicity

The cytotoxicity test of simvastatin, PMSs, and SIM/PMSs was evaluated in tenocytes. Tenocytes (5 × 10^4^ cells/well) were seeded in 96-well tissue culture plates containing simvastatin (1 mM), simvastatin (5 mM), 10 mg of PMSs, SIM (1 mM)/PMSs, and SIM (5 mM)/PMSs and maintained with DMEM supplemented with 10% FBS and 1% antibiotics. After 1 day or 2 days, the medium was aspirated and CCK-8 proliferation reagents were added. According to the manufacturer’s instructions, the cells were further incubated for 1 h at 37 °C. The optical density of the live cells was measured using a Flash Multimode Reader at 450 nm.

### 4.6. In Vitro Anti-Inflammatory Effects of SIM/PMSs on Inflamed Tenocytes

The in vitro anti-inflammatory effects of SIM/PMSs were tested using tenocytes that were isolated from the Achilles tendons of Sprague Dawley (SD) rats (8-week-old males, DooYeol Biotech, Seoul, Korea). The tenocyte isolation was performed as previously described [21]. The experimental protocol for this isolation was approved by the Institutional Animal Care and Use Committee of the Korea University Medical Center (KOREA-2016-0250, 29 November 2016).

In order to demonstrate the in vitro anti-inflammatory effects of SIM/PMSs on LPS-treated tenocytes, we analyzed the *mRNA* levels of *MMP-3*, *COX-2*, *IL-6*, and *TNF-α* using real-time PCR. Tenocytes (1 × 10^5^ cells/mL/well) were carefully seeded on the PMSs (10 mg) or SIM (1 mM or 5 mM)/PMSs in 24-well tissue culture plates. After 24 h of incubation, LPS (1 μg/mL) was applied to the tenocytes in all groups. After three days, the cells were harvested. The total RNA was isolated from cells in each group using an RNeasy Plus Mini Kit (Qiagen, Valencia, CA, USA) according to the manufacturer’s instructions. The total RNA (1 μg) was reverse-transcribed into cDNA using AccuPower RT PreMix (Bioneer, Daejeon, Korea) according to the manufacturer’s instructions. The primers and PCR conditions used for amplification of *MMP-3*, *COX-2*, *IL-6*, and *TNF-α* are as follows: *MMP-3*; (F) 5′-ACC TGT CCC TCC AGA ACC TG-3′, (R) 5′-AAC TTC ATA TGC GGC ATC CA-3′, *COX-2*; (F) 5′-CAG CCA TAC TAT GCC TCG GA-3′, (R) 5′-GGA TGT CTT GCT CGT CGT TC-3′, *IL-6*; (F) 5′-CCG TTT CTA CCT GGA GTT TG-3′, (R) 5′-GTT TGC CGA GTA GAC CTC AT-3′, and *TNF-α*; (F) 5′-CTC CCA GAA AAG CAA GCA AC-3′, (R) 5′-CGA GCA GGA ATG AGA AGA GG-3′. PCR amplification and detection were performed using an ABI7300 Real-Time Thermal Cycler (Applied Biosystems, Foster, CA, USA). The relative *mRNA* levels of *MMP-3*, *COX-2*, *IL-6*, and *TNF-α* were normalized to those of glyceraldehyde 3-phophate dehydrogenase (*GAPDH*).

### 4.7. Animal Models of Collagenase-Induced Achilles Tendinitis

We established an Achilles tendinitis animal model (using collagenase injection) to demonstrate the in vivo anti-inflammatory and tendon healing effects of SIM/PMSs, as described in our previous study [21]. The in vivo animal experiments were performed and approved by the Institutional Animal Care and Use Committee of the Korea University Medical Center (KOREA-2016-0250, 29 November 2016). SD rats (8-week-old males, DooYeol Biotech, Seoul, Korea) were anesthetized with isoflurane (1% *w*/*v* in 2 L oxygen). The rats received a single injection of 50 μL of collagenase type I in the right Achilles tendon (Col I) (50 mg/mL inn PBS (pH 7.4)). Seven days after the collagenase injection, 10 mg of the microspheres were mixed with 1 mL of 2% carboxymethyl cellulose (CMC) solution. Randomly selected rats received an injection of 50 μL of CMC solution of the microspheres. The real treatment dosages of simvastatin in each SIM/PMS group were 2.39 μg/rat for SIM (1 mM)/PMSs and 12.18 μg/rat for SIM (5 mM)/PMSs. Simvastatin (105 μg/rat) was used as the drug control, and was injected into the Achilles tendon of control animals. Six weeks after treatment with PMSs, simvastatin, SIM (1 mM)/PMSs, and SIM (5 mM)/PMSs, the rats were euthanized for further analysis. The rats were divided into the following six groups: (I) control (no treatment), (II) Col (I) (collagenase treatment), (III) Col (I) + PMSs, (IV) Col (I) + simvastatin, (V) Col (I) + SIM (1 mM)/PMSs, and (VI) Col (I) + SIM (5 mM)/PMSs.

### 4.8. Histopathological Evaluations

The calcaneus-Achilles tendon specimens were harvested from the sacrificed rats and fixed in 3.7% (*v*/*v*) paraformaldehyde for histological evaluations. The specimens were then dehydrated in ethanol and embedded in paraffin. The tissue blocks were sectioned longitudinally in the rotary microtome (HM 355S automatic microtomes, Thermo Scientific, Waltham, MA, USA) at 5 μm thickness. These sliced tissue samples were stained with Masson’s trichrome.

### 4.9. Biomechanical Test

The tendon specimens were fixed to a specially designed device, which allowed the specimen to be oriented such that a tensile load could be applied along the axis of the tendon. The fixed specimen device was tested on an Instron Mechanical Tester (AG-10KNX, Shimadzu, Kyoto, Japan) at a cross-head speed of 5 mm/min. A 1 newton load cell was used to measure the loading force. The ultimate tensile strength (defined as maximum stress or force per unit area) and stiffness (force required per unit displacement) were also obtained.

### 4.10. Hydroxyproline Assay

In order to investigate the Achilles tendon healing effects of SIM/PMSs, the collagen contents of Achilles tendon tissues in each group were demonstrated by measuring the hydroxyproline assay. In brief, 5 mg of Achilles tendon tissue was hydrolyzed with 6 normal HCl under heating at 110 °C for 12 h. The HCl was evaporated at 60 °C, and the hydrolyzed tissues were reconstituted in DW (20 mL) and neutralized with NaOH to pH 7.0. Chloramine T solution (50 μL, 60 mM) was mixed with 100 μL of standard hydroxyproline or samples in a 96-well plate to initiate hydroxyproline oxidation. After 20 min of incubation at room temperature (RT), the chloramine T was destroyed by adding 50 μL of perchloric acid (HClO_4_) to each well. The absorbance was read using a Flash Multimode Reader (Thermo Scientific, San Jose, CA, USA) at 550 nm.

### 4.11. In Vivo Anti-Inflammatory Effects of SIM/PMSs on Collagenase-Induced Achilles Tendinitis

The in vivo anti-inflammatory effects of SIM/PMSs on Achilles tendinitis were investigated by analyzing the *gene* expressions of pro-inflammatory cytokines (*MMP-3*, *COX-2*, *IL-6*, *TNF-α*, and *MMP-13*) and anti-inflammatory cytokines (*IL-4*, *IL-10*, and *IL-13*) using real-time PCR. The *gene* expression levels of these pro- and anti-inflammatory cytokines were determined using the total RNA extracted from the whole blood in each group. The whole blood was collected from an ear vein of a rat in each group at weeks: 0 (no treatment), 1 (1 week after collagenase treatment, 2 (2 weeks after collagenase treatment and 1 week after treatment of SIM or PMSs with or without SIM), and 6 (7 weeks after collagenase treatment and 6 weeks after treatment of SIM or PMSs with or without SIM). The total RNA was extracted from the collected whole blood samples at the pre-determined time points using a QlAamp RNA Blood Mini Kit (Qiagen, Valencia, CA, USA). The total RNA concentration in each group was measured using a Nanodrop spectrometer (ND-1000 spectrometer, NanoDrop Technologies Inc., Wilmington, DE, USA). The total RNA (1 μg) was reverse-transcribed into cDNA. The same primer sequences for the pro-inflammatory cytokines (including *MMP-3*, *COX-2*, *IL-6*, and *TNF-α genes*) were used as described above for the in vitro anti-inflammatory studies. The primer sequence of *MMP-13* was as follows: *MMP-13*; (F) 5’-AAG GAG CAT GGC GAC TTC TA-3’, (R) 5’-GGT CCT TGG AGT GGT CAA GA-3’. The primer sequences of the anti-inflammatory cytokines, including *IL-4*, *IL-10*, and *IL-13*, were as follows: *IL-4*; (F) 5’-ACA GGA GAA GGG ACG CCA T-3’, (R) 5’-GAA GCC CTA CAG ACG AGC TCA-3’, *IL-10*; (F) 5’-GGT TGC CAA GCC TTA TCG GA-3’, (R) 5’-ACC TGC TCC ACT GCC TTG CT-3’ and *IL-13*; (F) 5’-AGA CCA GAC TCC CCT GTG CA-3’, (R) 5’-TGG GTC CTG TAG ATG GCA TTG-3’. PCR amplification and detection was performed using a ABI7300 Real-Time Thermal Cycler (Applied Biosystems) using a DyNAmo^TM^ SYBR^®^ Green qPCR Kit (Finnzymes, Espoo, Finland). The relative *gene* expression levels of pro-inflammatory cytokines (*MMP-3*, *COX-2*, *IL-6*, *TNF-α*, and *MMP-13*) and anti-inflammatory cytokines (*IL-4*, *IL-10*, and *IL-13*) were normalized to those of *GAPDH*.

### 4.12. Statistical Analysis

Data are presented as means ± standard deviations. Statistical comparisons were carried out via one-way analysis of variance (ANOVA) using Systat software ver. 13 (SigmaPlot, IL, USA). Differences were considered statistically significant at * *p* < 0.05 and ** *p* < 0.01.

## 5. Conclusions

We used a simple fluidic device to develop a long-term simvastatin delivery system using porous microspheres. The SIM/PMSs are capable of long-term simvastatin delivery in a sustained manner. This treatment effectively and dose-dependently decreased the *mRNA* levels of pro-inflammatory cytokines in the LPS-treated tenocytes. In vivo studies demonstrated that SIM/PMSs significantly decreased the levels of pro-inflammatory cytokines, while they markedly increased anti-inflammatory cytokine levels. These anti-inflammatory effects of SIM/PMSs may ultimately allow improved tendon healing and restoration by increasing the collagen content, stiffness, and the tissue’s overall tensile strength.
